# Epiisopiloturine, an Alkaloid from *Pilocarpus microphyllus*, Attenuates LPS-Induced Neuroinflammation by Interfering in the TLR4/NF-*κ*B-MAPK Signaling Pathway in Microglial Cells

**DOI:** 10.1155/2023/4752502

**Published:** 2023-04-28

**Authors:** João Antônio Costa de Sousa, Francisco Vinícius Clemente Serra Azul, Ana Bruna de Araújo, Rebeca Colares Tomé, Francisca Raysse Mesquita Silva, Silvânia Maria Mendes de Vasconcelos, Francisco José Rios, Luzia Kalyne Almeida Moreira Leal

**Affiliations:** ^1^Center of Cosmetics and Pharmaceutical Studies, CEFAC, Faculty of Pharmacy, Odontology, and Nursing, Department of Pharmacy, Federal University of Ceará, CEFAC, Fortaleza, CE, Brazil; ^2^Neuropharmacology Laboratory, Department of Physiology and Pharmacology, NPDM, Federal University of Ceará, Fortaleza, CE, Brazil; ^3^Institute of Cardiovascular and Medical Sciences, University of Glasgow, 126 University Place, Glasgow G12 8TA, UK

## Abstract

Neuroinflammation is present in the pathophysiological mechanisms of several diseases that affect the central nervous system (CNS). Microglia have a prominent role in initiating and sustaining the inflammatory process. Epiisopiloturine (EPI) is an imidazole alkaloid obtained as a by-product of pilocarpine extracted from *Pilocarpus microphyllus* (jaborandi) and has shown promising anti-inflammatory and antinociceptive properties. In the present study, we investigated the effects of EPI on the inflammatory response in microglial cells (BV-2 cells) induced by lipopolysaccharide (LPS) and explored putative underlying molecular mechanisms. Cell viability was not affected by EPI (1-100 *μ*g/mL) as assessed by both LDH activity and the MTT test. Pretreatment with EPI (25, 50, and 100 *μ*g/mL) significantly reduced the proinflammatory response induced by LPS, as observed by a decrease in nitrite oxide production and iNOS protein expression. EPI (25 *μ*g/mL) reduced IL-6 and TNF-*α* production, by 40% and 34%, respectively. However, no changes were observed in the anti-inflammatory IL-10 production. Mechanistically, EPI inhibited the TLR4 expression and phosphorylation of NF-*κ*B p65 and MAPKs (JNK and ERK1/2) induced by LPS, but no changes were observed in TREM2 receptor expression in LPS-stimulated cells. In conclusion, our data demonstrated the potent anti-inflammatory properties of EPI in microglial cells. These effects are associated with the reduction of TLR4 expression and inhibition of intracellular signaling cascades, including NF-*κ*B and MAPKs (JNK and ERK1/2).

## 1. Introduction

Inflammatory response plays a crucial role in the development and progression of many neurodegenerative diseases (ND) that are closely related to aging, such as Parkinson's disease (PD) and Alzheimer's disease (AD), which are progressive chronic illnesses and result in patient death [1–3]. However, the current pharmacotherapy approved by the Food and Drug Administration is mostly symptomatic and has limitations. Hence, it is of high priority to investigate new drugs to prevent or improve the treatment of these diseases [4].

The microglial cells are considered “Central Nervous System (CNS) macrophages,” constituting 5–10% of total brain cells, and interact with almost all cell types in the brain (e.g., astrocytes, oligodendrocytes, and neurons) contributing to brain development, plasticity, and maintaining of the homeostasis [5]. Microglial cells modulate neuronal activity, and in return, neurons may also interfere with microglial functions. Under stress conditions, microglia might acquire different activation phenotypes that vary according to the microenvironment including (i) classically activated M1 phenotype characterized by the production of proinflammatory mediators, including IL-1*β*, TNF-*α*, and IL-6, and increased expression of inducible nitric oxide synthase (iNOS) and surface markers, CD16/32, CD86, and CD40), and (ii) alternatively activated M2 phenotype, which is associated with phagocytosis of apoptotic cells and release of anti-inflammatory mediators (IL-10, TGF*β*, and galectin-3) [6–8]. In ND, there is an imbalance of M1/M2 favoring M1.

In the CNS, microglia interact with damage-associated molecular patterns (DAMPS) (cell debris, modified proteins, including A*β* species and alpha-synuclein, and oxidized lipids) or pathogen-associated molecular patterns (PAMPs) such as LPS, a component of the outer membrane of Gram-negative bacteria. This PAMP acts by activating the innate immune receptor Toll-like 4 (TLR4) which is highly expressed in microglia. The LPS activates intracellular signaling pathways associated with transcription factors such as NF-*κ*B dimer (p50 and p65) and mitogen-activated protein kinases (MAPKs), producing inflammatory mediators. In contrast, the triggering receptors expressed on myeloid cells 2 (TREM 2) and expressed in microglial cells have opposite effects by downregulating the production of proinflammatory mediators, such as TNF-*α* [9–11].

The activation of NF-*κ*B by LPS-TLR4 and subsequent translocation of this transcription factor from the cytoplasm to the nucleus leads to transcription of genes involved in the production of inflammatory enzymes (e.g., iNOS), TNF-*α*, IL-1*β*, IL-6, and other chemical mediators, while the activation of MAPK family members such as JNK, ERK1/2, and p-38 results in the activation of activator protein 1 (AP-1) family transcription factors which regulate the expression of various inflammatory proteins [9, 12–15]. Thus, several studies have been assessing the modulation of microglial functions as a strategy to treat ND. The therapeutic approach is associated with microglial activation, by targeting innate immune receptors and intracellular signaling pathways including TLR, MAPKs, and transcription factors that are directly involved in the production of neuroinflammatory mediators and oxidant species (e.g., nitric oxide and reactive oxygen species) associated to ND [16–18]. Of importance, various plant-derived alkaloids, such as berberine and caffeine, have shown neuroprotective effects by reducing neuroinflammation and regulating microglial cell functions [19].

**Figure 1:**
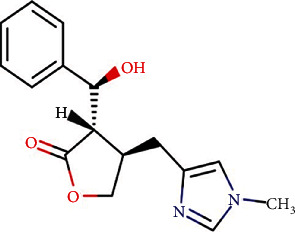
Chemical structure of epiisopiloturine (EPI) from *Pilocarpus microphyllus.*

Epiisopiloturine (EPI) (see Figure 1) is an imidazole alkaloid obtained as a by-product of pilocarpine extracted from *Pilocarpus microphyllus*. Recently, we demonstrated that EPI reduces the inflammatory response in human neutrophils by suppressing NF-*κ*B activation, TNF*α*, IL-6, and ROS production [20]. We also demonstrated an antinociceptive effect of this alkaloid in carrageenan-induced acute inflammatory pain models in rodents [20, 21]. Additional pharmacological activities have been described for EPI including antibacterial, antiproliferative, anti-inflammatory, and antinociceptive in mice [22–24]. In the current study, we investigated the effects of EPI on the inflammatory response induced by LPS on the microglia cell line. In particular, we focused on cytokine production and phosphorylation of MAPK and NF-*κ*B pathways.

## 2. Materials and Methods

### 2.1. Alkaloid Epiisopiloturine

The epiisopiloturine alkaloid (EPI) (99.7% purity) was obtained as a by-product of pilocarpine extracted from *P. microphyllus*, and a voucher specimen (HDELTA2869) was deposited in the herbarium Delta do Parnaíba, Federal University of Piauí. EPI was dissolved in dimethyl sulfoxide (DMSO) 0.1% in water.

### 2.2. Chemicals and Biological Materials

N-(1-Naphthyl)ethylenediamine dihydrochloride (NEED) (Sigma-Aldrich, EUA), bovine serum albumin (BSA) (Sigma-Aldrich, EUA), absolute ethyl alcohol (Dinâmica, Brazil), 3-(4,5-dimethylthiazol-2-yl)-2,5-diphenyltetrazolium bromide (MTT) (Sigma-Aldrich, EUA), sodium chloride (Dinâmica, Brazil), potassium chloride (Dinâmica, Brazil), DMSO (Sigma-Aldrich, EUA), sodium dodecyl sulfate (SDS) (Biorad, EUA), sodium deoxycholate (Bio-Rad, EUA), monobasic sodium phosphate (Dinâmica, Brazil), dibasic sodium phosphate (Dinâmica, Brazil), glycerol (Dinâmica, Brazil), lipopolysaccharide (LPS) (Sigma-Aldrich, EUA), methanol (Dinâmica, Brazil), Roswell Park Memorial Institute (RPMI) (Gibco by Life Technologies, EUA), fetal bovine serum (FBS) (Gibco by Life Technologies, EUA), sulfanilamide (Dinâmica, Brazil), Tris-HCl (Dinâmica, Brazil), Triton x-100 (Sigma-Aldrich, EUA).

Fetal bovine serum (FBS) and cell culture medium RPMI-1640 were purchased from Gibco, USA. Murine microglial cells (BV-2 cell line) were acquired from Cell Biobank, Rio de Janeiro, Brazil. Anti-TLR4 and anti-TREM2 were obtained from Invitrogen, Carlsbad, CA, USA. Anti-p-P65 NF-*κ*B, anti-p-NF-*κ*B total, anti-p-JNK, anti-p-total JNK, and anti-p-ERK1/2 were obtained from Cell Signaling Technology®, Danvers, MA, USA. Anti-ERK1/2 total was obtained from Abcam®, Cambridge, and anti-mouse secondary antibody was obtained from Bio-Rad Laboratories, CA, USA, or anti-rabbit was obtained from Sigma-Aldrich, New York, USA.

### 2.3. Cell Culture Conditions

Cells were plated on poly-L-lysine-coated 96- or 24-well plates, at a density of 1 × 10^5^ cells/mL or 1 × 10^6^ cells/mL in RPMI-1640 culture medium, supplemented with 10% FBS at 37°C under an atmosphere of 5% CO_2_.

### 2.4. MTT Assay

BV-2 cells (1 × 10^5^ cells/mL) were incubated with different concentrations of EPI (1, 10, 25, 50, and 100 *μ*g/mL), vehicle/control group (DMSO 0.1% in water), or Triton x-100 (0.01%—cytotoxic standard). After 24 hours, the MTT bromide salt (3[4,5-dimethylthiazol-2-yl]-2,5-diphenyltetrazolium bromide) was added to cell suspension at a concentration of 0.5 mg/mL. After 90 minutes of incubation, the plate was centrifuged at 800 g and the supernatant was discarded. Finally, 150 *μ*L of DMSO was added for solubilization of the metabolized formazan salt. The plate was stirred constantly for 15 minutes using a shaker, and the absorbance was measured in a microplate reader at 560 nm [25].

### 2.5. Lactate Dehydrogenase Activity

The assay was performed using the LDH Kit (Liquiform, Minas Gerais, Brazil) according to the manufacturer's instructions. The assay is based on the measurement of the decrease in absorbance of the sample due to oxidation of nicotinamide adenine dinucleotide (NADH), which is proportional to the LDH activity. Microglial cells BV-2 (1 × 10^5^ cells/mL) were plated in 96-well plate and after 1 hour were incubated with EPI diluted in DMSO 0.1% (1, 10, 25, 50, and 100 *μ*g/mL), vehicle/control group (DMSO 0.1%), or Triton x-100 group (0.01%—cytotoxic standard). After 24 hours, the substrate of the LDH enzyme was added to cell supernatants. Absorbance (340 nm) was measured at 37°C.

### 2.6. NO Measurement

Nitric oxide (NO) production was assessed by nitrite concentration using the Griess reaction. Microglial BV-2 cells (1 × 10^6^ cells/mL) were incubated with EPI (1, 10, 25, 50, and 100 *μ*g/mL) or vehicle/control group (DMSO 0.1%) for one hour, followed by LPS stimulation (0.5 *μ*g/mL). Next, 100 *μ*L of Griess reagent (1% sulfanilamide in 1% H_3_PO_4_/0.1% N-(1-naphthyl)-ethylenediamine dihydrochloride/1% H_3_PO_4_/distilled water, 1 : 1 : 1 : 1) was added to 100 *μ*L of cell culture supernatant. Absorbance was measured at 560 nm using a plate reader. Nitrite concentration was obtained from the absorbance values using sodium nitrite (1 *μ*M - 100 *μ*M) as the standard curve [26].

### 2.7. Measurement of IL-1*β*, IL-6, TNF-*α*, and IL-10 Levels

Cytokine production was assessed by enzyme-linked immunosorbent assay (ELISA). BV-2 cells (1 × 10^6^ cells/mL) were pretreated with EPI, 25 *μ*g/mL, or vehicle/control group (DMSO 0.1%) for one hour at 37°C, followed by LPS stimulation (0.5 *μ*g/mL). After 24 hours, IL-1*β*, IL-6, IL-10, and TNF-*α* were measured in the cell supernatant according to the manufacturer's instructions (BD Biosciences—San Diego, CA, USA, and R&D Systems—Minnesota, MN, USA). Optical density was measured at an absorbance of 450 nm using a microplate reader.

### 2.8. Western Blot Analysis

Microglial cells (1 × 10^6^ cells/mL) were incubated with EPI 25 *μ*g/mL or vehicle/control group (DMSO 0.1%) for one hour at 37°C, followed by LPS stimulation (0.5 *μ*g/mL) for 1 h or 24 h. After the incubation period, the cells were harvested with ice-cold buffered saline (PBS) and centrifuged at 4°C, 130 g for 5 minutes. The total cell lysate was obtained using radioimmunoprecipitation assay buffer (RIPA buffer) supplemented with protease inhibitors (P2714, Sigma-Aldrich, New York, USA) and phosphatase inhibitors (ab201112, Abcam®, Cambridge, UK). The total lysate was cleared by centrifugation at 12000 g for 10 minutes at 4°C, and the pellet was discarded. Protein concentration was determined using the BCA kit (Bio-Rad Laboratories, CA, USA). Total protein (30 *μ*g) was separated on 10% SDS-polyacrylamide (SDS-PAGE) gel electrophoresis and transferred onto a polyvinylidene fluoride (PVDF) membrane (Bio-Rad Laboratories, CA, USA). Nonspecific binding sites were blocked with 5% nonfatty dry milk solubilized in Tris-buffered saline solution with Tween 0.01% for 1 h at room temperature. Membranes were incubated overnight at 4°C with the following primary specific antibodies: anti-iNOS 1 : 3000 (ab178945), anti-*β*-actin 1 : 100 (ab8226), and anti-total-ERK1/2 total 1 : 1000 (ab36991) from Abcam; anti-TLR4 1 : 500 (482300) and anti-TREM2 1 : 500 (PA587933) from Thermo Scientific; anti-phospho-NF-*κ*B P65 1 : 1000 (Ser536, #3033S), anti-total-NF-*κ*B P65 1 : 1000 (#6956S), anti-phospho-JNK 1 : 1000 (Thr183/Tyr185, #9255S), anti-total-JNK 1 : 2000 (#9252S), and anti-phospho-ERK1/2 1 : 1000 (Thr202/Tyr204, #9101S) all from Cell Signaling. Next, membranes were washed with TBS-tween and incubated with secondary antibodies anti-mouse (#170-6516, Bio-Rad) or anti-rabbit (SAB3700956, Sigma-Aldrich) (HRP 1 : 3000) for 2 hours. Protein expression was visualized using Western Blot Clarity™ ECL (Bio-Rad Laboratories, Hercules, CA, USA). Immunoreactive signals were detected by chemiluminescence in a photo documenter ChemiDocTM MP Imaging System (Bio-Rad Laboratories, Hercules, CA, USA). Optical densities were measured by Image Lab™ 5.1 Software (Bio-Rad Laboratories, Hercules, CA, USA).

### 2.9. Statistical Analyses

For statistical analysis, the GraphPad Prism version 6.0 program (GraphPad Software, San Diego, USA) was used. Data were represented as mean ± standard error of the mean (S.E.M). Data normality analysis was performed using the Shapiro-Wilk test. Data that showed normal distribution were analyzed by analysis of variance (one-way or two-way ANOVA) followed by the Bonferroni posttest. Differences were considered significant when *p* < 0.05.

## 3. Results

### 3.1. Cell Viability Is Not Affected by EPI

**Figure 2:**
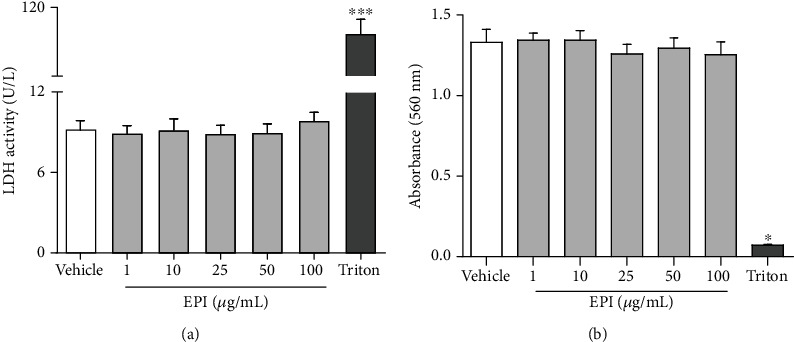
Effect of EPI on cell viability. LDH activity (a) and MTT test (b) were assessed in microglial BV-2 cells treated with different concentrations of EPI. Results are expressed as mean ± S.E.M. ^∗^ vs. vehicle/RPMI (*p* < 0.05—one-way ANOVA and Bonferroni test), *n* = 4/group.

The addition of increasing concentrations of EPI (1, 10, 25, 50, and 100 *μ*g/mL) to BV-2 cells did not affect cell viability assessed LDH enzyme activity (8.8 ± 0.5; 9.1 0.8; 8.8 ± 0.6; 8.9 ± 0.6, and 9.8 ± 0.6 U/L, respectively) compared to vehicle group (DMSO 0.1%: 9.1 ± 0.7 U/L) (Figure 2(a)). In the evaluation of cell viability by MTT, the addition of increasing concentrations of EPI (1, 10, 25, 50, and 100 *μ*g/mL) to the BV-2 cell suspension did not significantly reduce cell viability (Abs 560 nm: 1.3 ± 0.04; 1.3 ± 0.05; 1.2 ± 0.05; 1.2 ± 0.06; 1.2 ± 0.06, respectively) when compared to the vehicle group (Abs 560 nm: 1.3 ± 0.08) (Figure 2(b)). These results demonstrated that EPI does not affect the viability of microglial cells and guided the continuation of studies to investigate the anti-inflammatory/immunomodulatory effect of this alkaloid in BV-2 cells.

### 3.2. EPI Reduces Nitric Oxide/Nitrite Production in LPS-Stimulated BV-2 Cells

**Figure 3:**
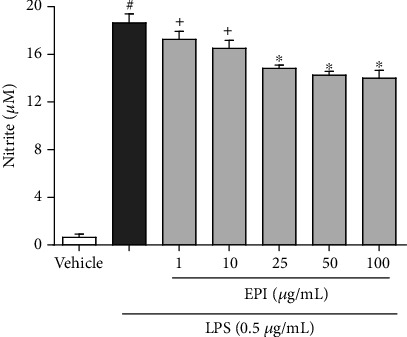
Effect of EPI in LPS-induced NO production. Results are expressed as mean ± S.E.M of nitrite production (*μ*M). # vs. vehicle/RPMI; ^∗^ vs. LPS group. + vs. EPI 25 *μ*g/mL+LPS (*p* < 0.05—one-way ANOVA and Bonferroni test), *n* = 4/group.

Microglial cells treated with LPS (0.5 *μ*g/mL) exhibited higher concentrations of nitrite in the supernatant (18.6 ± 0.7 *μ*M) compared to the vehicle control group (DMSO 0.1%: 0.6 ± 0.2 *μ*M) (Figure 3). With LPS treatment, the addition of EPI (25, 50, and 100 *μ*g/mL) significantly reduced nitrite levels (14.8 ± 0.2; 14.2 ± 0.3; 14.0 ± 0.6 *μ*M, respectively) when compared to only LPS-treated group (18.6 ± 0.7 *μ*M). Lower concentrations of EPI (1 and 10 *μ*g/mL) were not able to reduce nitrite levels (17.9 ± 0.2; 16.5 ± 0.6 *μ*M, respectively) induced by LPS stimulation. This finding shows a potential anti-inflammatory effect of EPI, which may be related to the inhibition of intracellular pathways such as iNOS, NF-*κ*B, and MAPKs.

### 3.3. EPI Reduces the Expression of Inducible Nitric Oxide Synthase (iNOS) and the Production of IL-1 *β*, IL-6, and TNF-*α* in Microglial Cell Stimulated with LPS

**Figure 4:**
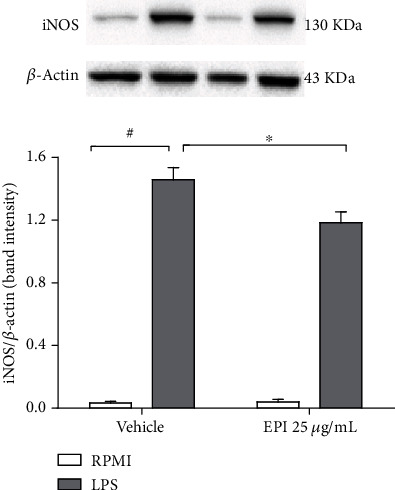
Effect of EPI in LPS-induced iNOS expression in microglia. BV-2 cells were pretreated with EPI (25 *μ*g/mL) for 1 h before the addition of LPS (0.5 *μ*g/mL). Results are expressed as mean ± S.E.M of iNOS/*β*-actin band intensity. # vs. vehicle/RPMI; ^∗^ vs. LPS group (*p* < 0.05—two-way ANOVA and Bonferroni test), *n* = 4/group.

Microglial cells treated with LPS (0.5 *μ*g/mL) exhibited increased expression of nitric oxide synthase. However, the pretreatment of cells with EPI (25 *μ*g/mL) (iNOS/*β*-actin band intensity: 1.1 ± 0.06) significantly reduced when compared to the LPS group (iNOS/*β*-actin band intensity: 1.4 ± 0.07) (Figure 4).

**Figure 5:**
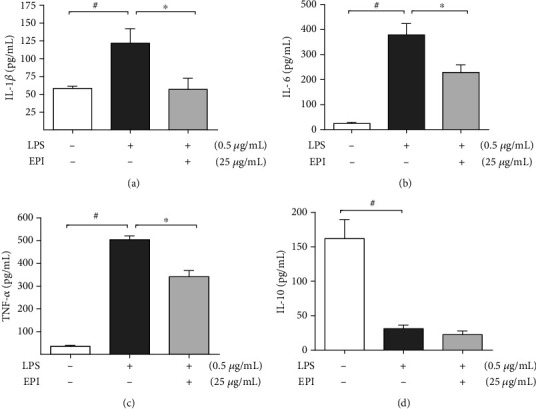
Effects of EPI on inflammatory cytokine release in LPS-stimulated microglia. BV-2 cells were pretreated with EPI (25 *μ*g/mL) for 1 h and incubated with LPS (0.5 *μ*g/mL) for 24 hours. Results are expressed as mean ± S.E.M of IL-1*β* (a), IL-6 (b), TNF-*α* (c), and IL-10 (d) production (pg/mL). # vs. vehicle/RPMI; ^∗^ vs. LPS group (*p* < 0.05—one-way ANOVA and Bonferroni test), *n* = 4/group.

Based on the results obtained so far, the concentration of EPI selected for further studies was 25 *μ*g/mL. Cells exposed to LPS showed an increase about two times in IL-1*β* concentration (121.0 ± 20.8 pg/mL). Pretreatment with EPI significantly reduced IL-1*β* production (64.7 ± 17.7 pg/mL) when related to the LPS group, with final values similar to those unstimulated BV-2 cells (58.3 ± 3.0 pg/mL) (Figure 5(a)). The addition of EPI (25 *μ*g/mL) to the cell culture before LPS also significantly reduced IL-6 production (228.4 ± 30.6 pg/mL) showing an average inhibition of 40% when compared to the LPS group (378.7 ± 44.9 pg/mL). Similarly, the increase of TNF-*α* production observed after exposure of the cells to LPS (504.2 ± 16.9 pg/mL) was partially reversed by EPI (341.5 ± 27.7 pg/mL) corresponding to a reduction of about 34% (see Figures 5(a) and 5(c)). It was also evaluated the effect of EPI on the production of IL-10, an anti-inflammatory cytokine. The cells exposed to LPS reduced in about five times the IL-10 production (31.2 ± 5.0 pg/mL) when related to unstimulated BV-2 cells (162.0 ± 27.6 pg/mL), and EPI (22.5 ± 5.2 pg/mL) did not interfere in the reduction of this cytokine level after exposure to LPS (Figure 5(d)). Taken together, these data show that EPI acts decreasing NO production through direct action on the biosynthesis of this mediator. In addition, the anti-inflammatory effect of this alkaloid seems to be associated to the reduction in the production of proinflammatory cytokines without affecting IL-10 level, an anti-inflammatory cytokine.

### 3.4. EPI Interferes with TLR4/NF-*κ*B-MAPK Signaling Pathway

**Figure 6:**
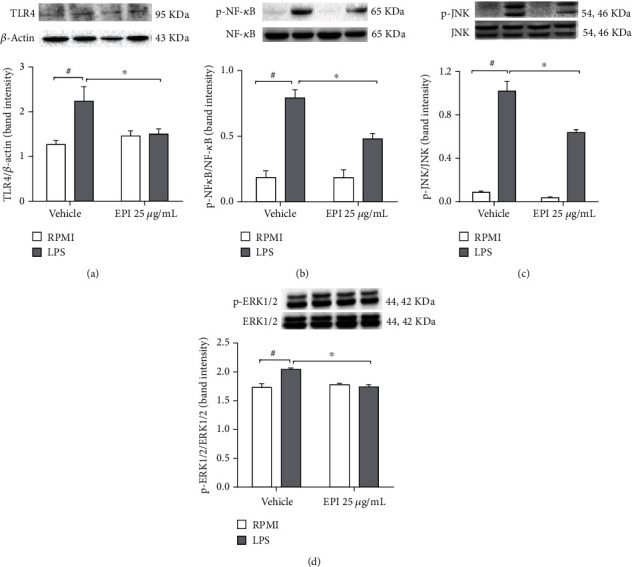
Effect of EPI on TLR4 (a), NF-*κ*B (b), JNK (c), and ERK1/2 (d). BV-2 cells were pretreated with EPI (25 *μ*g/mL) for 1 h, followed by LPS (0.5 *μ*g/mL) for 1 hour. Results are expressed as mean ± S.E.M. # vs. vehicle/RPMI; ^∗^ vs. vehicle group stimulated with LPS (*p* < 0.05—two-way ANOVA and Bonferroni test), *n* = 4/group.

Stimulation of microglial cells with LPS (0.5 *μ*g/mL) for 1 hour increased about two times the TLR4 protein expression (2.2 ± 0.3) when compared to the vehicle group (1.26 ± 0.09). However, pretreatment with EPI (25 *μ*g/mL), significantly reduced the protein expression of TLR-4 (1.3 ± 0.1) when related to the LPS group (Figure 6(a)). The addition of EPI (25 *μ*g/mL) to the cells inhibited in about 40% the phosphorylation of the p65 subunit of NF-*κ*B (0.47 ± 0.04) when compared to the LPS group (0.79 ± 0.06). Similar results were observed in the activation of the MAPK signaling pathway. EPI pretreatment reduced phosphorylation of JNK (0.63 ± 0.01) and ERK1/2 (1.74 ± 0.03) compared to the LPS-treated group (1.0 ± 0.01 and 1.74 ± 0.03 for JNK and ERK1/2, respectively) (Figures 6(c) and 6(d)). These data demonstrate that the anti-inflammatory effect of EPI occurs via the inhibition of TLR4/NF-*κ*B-MAPK (ERK and JNK) signaling pathway in BV-2 microglial cells, which affect the production of inflammatory mediators.

### 3.5. EPI Does Not Affect the Expression of Triggered Receptor Expressed on Myeloid Cells 2 (TREM2)

**Figure 7:**
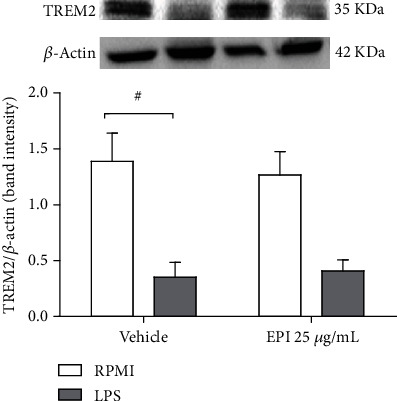
Expression of TREM in BV-2 cells treated with EPI. BV-2 cells were pretreated with EPI 25 *μ*g/mL for 1 h, followed by stimulation with LPS for 1 h. Results are expressed as mean ± of TREM2/*β* − actin band intensity. # vs. vehicle/RPMI; (*p* < 0.05—two-way ANOVA and Bonferroni test), *n* = 4/group.

The addition of LPS (0.5 *μ*g/mL) to BV-2 microglial cell suspension significantly reduced the protein expression of the anti-inflammatory marker TREM2 (0.35 ± 0.1) when compared to the vehicle group (1.3 ± 0.2). However, the cell treatment with EPI 25 *μ*g/mL (0.4 ± 0.2) was not able to significantly interfere with the receptor expression after LPS stimulation (0.3 ± 0.1) (see Figure 7). This data suggests, therefore, that the anti-inflammatory effect of EPI is not related to a modulatory effect in the anti-inflammatory mechanism mediated by the TREM2 receptor.

## 4. Discussion

This study investigates the anti-inflammatory effects of epiisopiloturine (EPI), an imidazole alkaloid isolated from *P. microphyllus* in LPS-stimulated microglia. Aberrant activation of microglia plays a crucial role in the development and progression of neuroinflammation responsible for progressive neuronal death in neurodegenerative diseases. The alkaloid EPI showed potent anti-inflammatory activity by reducing LPS-induced proinflammatory cytokines (M1 phenotype) through mechanisms associated with MAPK and NF-*κ*B signaling pathways.

A neuroinflammation is a form of inflammatory process restricted to the central nervous system (CNS) and peripheral nervous system (PNS), highly involved with several CNS diseases, including Parkinson's disease, multiple sclerosis, Alzheimer's disease (AD), and more recently neuropathic pain, due to the production of proinflammatory mediators (cytokines and chemokines) by glial cells [27–29]. Products of oxidative stress are among inflammatory mediators which contribute to the development and progression of these disorders, such as nitric oxide (NO).

Nitric oxide (NO) is a versatile mediator with several homeostatic roles, mainly in the cardiovascular and nervous systems; however, it is also produced in inflammatory processes by innate immune cells [30, 31]. In the central nervous system, NO production is associated with the activation of neuronal NO synthase (nNOS) and endothelial (eNOS), which are constitutively expressed in neurons of the spinal cord/vessels of the brain and vascular systems surrounding motor neurons, respectively. In contrast, the inducible isoform (iNOS) present in astrocytes and microglia is upregulated during inflammatory response [32]. Thus, during the inflammatory process in CNS, studies indicate that NO produced by iNOS contributes to neuronal death by mechanisms dependent on mitochondrial dysfunction, DNA damage, and release of glutamate with consequent excitotoxic cell death [33–37]. Here, we show that treatment of microglial cells with EPI reduced nitric oxide production and iNOS expression induced by LPS. In addition, these effects were not associated with cytotoxic effects of EPI as no changes were observed in the mitochondrial activity or cell membrane permeability assessed by the MTT test and LDH enzyme activity, respectively. These data suggest that EPI reduces NO production acting directly in its biosynthesis and might have consequences on the formation of other reactive species of nitrogen (RNS), such as peroxynitrite (ONOO-). At high concentrations, ONOO- in CNS induces lipid peroxidation, which affects the neuronal Ca^2+^ homeostasis, a molecular marker of AD. These data corroborate a previous study [38] showing that EPI reduced NO production and iNOS expression in intestinal tracts from rats affected by experimental Crohn's disease induced by trinitrobenzene sulfonic acid (TNBS).

The production of proinflammatory mediators (NO, IL-1*β*, TNF-*α*, and IL-6) by classically activated microglial cells (M1 profile) is a common process in neuroinflammation [39]. The chronic phenotype of this inflammatory profile (M1) downregulates the neurorepairing microglial profile (M2) responsible for restoring homeostasis in the brain parenchyma through the production of anti-inflammatory mediators such as IL-10, IL-4, TGF-*β*, and arginase-1 [40]. EPI reduced the increase in LPS-induced inflammatory cytokines in microglial cells. The higher IL-1*β* levels observed after exposure of the cells to LPS were reversed by EPI, which also prevented the TNF-*α* and IL-6 overproduction. Such data corroborate previous investigations where EPI was able to decrease the production of proinflammatory cytokines in human neutrophils [20], peritoneal cavity from mice submitted to carrageenan-induced peritonitis model [22], and intestinal inflammation in experimental TNBS-induced Crohn's disease [38].

Considering the promising results, the effectiveness of EPI in interfering with the TLR4 receptor expression induced by LPS in microglial cells was investigated. Toll-like receptors play important role in innate immune activation [16, 17]. They interact with pathogen-associated molecular patterns (PAMPs) including LPS and endogenous molecules (damage-associated molecular patterns (DAMPS)) leading to the expression of proinflammatory mediators [41]. LPS is a potent activation of TLR4, which is expressed in several cells from the immune, vascular, and central nervous system. TLR4 stimulation induces activation of a proinflammatory cascade involving adaptor molecules, activation of NF-*κ*B and MAPK pathways, and consequent cytokine production, including IL-1*β*, TNF-*α*, and IL-6. In CNS, TLR4 activation is associated with microglia-mediated neurotoxicity and it is considered therapeutic for ND treatment [42–47]. The EPI induced a significant reduction of the TLR4 expression, which explains the anti-inflammatory effect of this alkaloid. So, for further description of the mechanism of action of EPI, it was evaluated its effect on MAPK and NF-*κ*B pathways in BV-2 microglial cells.

NF-*κ*B and MAPKs are important intracellular activation pathways in the inflammatory response [48–55], and both pathways are potently activated by TLR4. EPI treatment inhibited the expression of the p65 NF-*κ*B subunit in microglial cells stimulated with LPS. The p50 and p65 dimer is the most frequent in the formation of NF-*κ*B and is found in an inactive form in the cytoplasm cells. The LPS-induced NF-*κ*B activation through TLR4 and adaptor molecules (MyD88 and TRIF) results in the phosphorylation of I*κ*B through the IKK complex. In the cell nucleus, the NF-*κ*B dimer initiates transcription of a number of different genes including chemokines, adhesion molecules, TNF-*α*, and IL-6 [56]. Thus, the result demonstrates that EPI reduced the production of inflammatory mediators by regulating the transcription of coding proinflammatory genes, such as IL-1*β,* TNF-*α,* and IL-6. Our results were corroborated by a previous study [20] where we demonstrated that this alkaloid also inhibits the migration of the p65 subunit to the nucleus in human neutrophils stimulated by fMLP. In addition, we show for the first time that the anti-inflammatory action of EPI is also dependent on its inhibitory effects on MAPK activation, particularly ERK 1/2 and JNK. The ERK and JNK signaling pathways control several cell functions, and JNK also acts in activating transcription factors, including AP-1, and activating transcription factor 2 (ATF-2) [57]. Studies have shown a key role of ERK 1/2 and JNK in a variety of physiological and pathological processes in CNS. Inhibitors of ERK and JNK have been studied as a pharmacological tool for the treatment of AD [58]. Thus, the regulation of these kinases by EPI makes it a promising drug for the treatment of AD.

**Figure 8:**
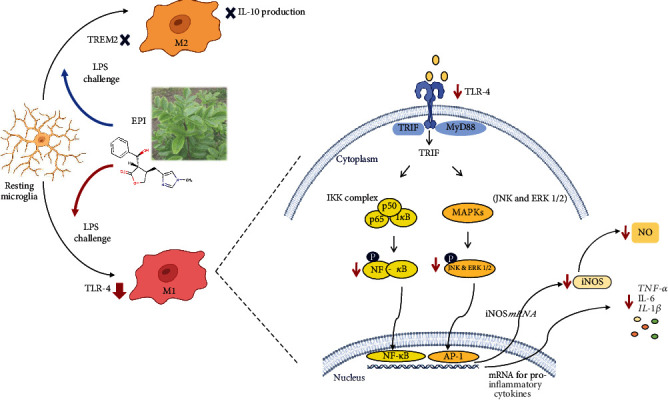
Anti-inflammatory effects mediated by the alkaloid epiisopiloturine (EPI) in microglia. EPI inhibits TLR4 expression and phosphorylation of NF-*κ*B p65 and MAPKs (JNK and ERK1/2) induced by LPS.

Taken together, our results indicate that EPI reduces neuroinflammatory response in two different manners: (1) reducing TLR4 expression and (2) interfering with downstream kinases (ERK 1/2 and JNK) and NF-*κ*B pathways (see Figure 8).

No other previous study had proposed this molecular mechanism of action for this alkaloid. Although several studies in the literature already highlight the ability of other alkaloids to inhibit similar intracellular signaling pathways in BV-2 cells, such as protopine obtained from *Corydalis yanhusuo* [59] and isotalatizidine from the species *Aconitum carmichaelii* Debx [60], both present in Chinese medicine.

TREM2 is a microglia-specific receptor for the M2-activation phenotype. The downregulation of this receptor contributes to the TLR4-mediated MAPK signaling pathway and increases A*β* protein accumulation and neuroinflammation in rodents [61–66]. Interestingly, EPI was not able to reverse the inhibition of TREM2 expression induced by LPS activation in BV-2 cells. These data suggest that EPI regulates the inflammatory response in microglial cells acting on TLR4, not affecting the expression of TREM2 (see Figure 8).

Under inflammatory conditions, activated microglia secrete various chemokines, resulting in leukocyte migration, e.g., neutrophils, which migrate from postcapillary venules to the brain, by crossing the blood-brain barrier. Activated neutrophils release various chemical mediators including cytokines, proteases, ROS, and RNS [67, 68]. Thus, the ability of EPI to regulate the proinflammatory mechanisms in microglial and human neutrophils as determined previously by our laboratory [20] makes EPI a potential therapeutic tool for the treatment of neuroinflammatory diseases by regulating central and peripheral innate immunity.

## 5. Conclusion

The present study demonstrates for the first time the anti-inflammatory activity of imidazole alkaloid epiisopiloturine (EPI) from *P. mycrophyllus* by inhibiting the LPS-induced inflammatory response in microglial cells. Mechanisms underlying these effects are associated with inhibition of TLR4 expression and signal transduction, interfering with downstream kinases (ERK1/2 and JNK), as well as NF-*κ*B pathway with consequent reduction of inflammatory mediators production (NO, IL-1 *β*, IL-6, TNF-*α*). Moreover, no effects were observed on TREM2 expression and IL-10 production.

## Data Availability

The experimental data used to support the findings of this study are included within the article.

## Abbreviations

AD: Alzheimer's disease
AP-1: Activator protein-1
BSA: Bovine serum albumin
CNS: Central nervous system
DMSO: Dimethyl sulfoxide
DNA: Deoxyribonucleic acid
ERK: Extracellular signal-regulated kinase
eNOS: Endothelial nitric oxide synthase
EPI: Epiisopiloturine
FBS: Fetal bovine serum
FMLP: N-formyl-methionyl-leucyl-phenylalanine
IFN-*γ*: Interferon-gamma
IKK: IkB kinase complex
IL: Interleukin
iNOS: Inducible nitric oxide synthase
JNK: c-Jun N-terminal kinase
LDH: Lactate dehydrogenase
LPS: Lipopolysaccharide
MAPKs: Mitogen-activated protein kinases
MTT: 3-(4,5-Dimethylthiazol-2-yl)-2,5-diphenyltetrazolium bromide
NADH: Nicotinamide adenine dinucleotide
NEED: N-(1-Naphthyl)ethylenediamine dihydrochloride
NF-*κ*B: Nuclear factor-kappa B
NO: Nitric oxide
ND: Neurodegenerative diseases
nNOS: Neuronal nitric oxide synthase
PAMPs: Pathogen-associated molecular patterns
PBS: Phosphate-buffered saline
PRRs: Pattern recognition receptors
RIPA: Radioimmunoprecipitation assay buffer
PMA: Phorbol 12-myristate-13-acetate
PNS: Peripheral nervous system
RNS: Reactive nitrogen species
ROS: Reactive oxygen species
SDS: Sodium dodecyl sulfate
TLR4: Toll-like receptor 4
TNF-ɑ: Tumor necrosis factor-alpha
TGF-*β*: Transformative growth factor *β*
TNBS: Trinitrobenzene sulfonic acid
TREM2: Receptor expressed on myeloid cells 2.
